# Optimization of the Equine-Sperm Freeze Test in Purebred Spanish Horses by Incorporating Colloidal Centrifugation

**DOI:** 10.3390/ani13030382

**Published:** 2023-01-22

**Authors:** Luna Gutiérrez-Cepeda, Francisco Crespo, Juan Carlos Blazquez, Consuelo Serres

**Affiliations:** 1 Animal Medicine and Surgery Department, Veterinary Faculty, UCM, Avda. Puerta de Hierro s/n, 28040 Madrid, Spain; 2 Centro Militar de Cría Caballar (CCFAA), C/Arsenio Gutiérrez Palacios s/n, 05005 Ávila, Spain

**Keywords:** equine sperm, freeze test, colloidal centrifugation, cryopreservation

## Abstract

**Simple Summary:**

The Purebred Spanish Horse, according to our clinical experience, is characterized by having a high number of stallions that do not meet the international commercial recommendations for equine-sperm cryopreservation. We investigated if the incorporation of single-layer colloidal centrifugation prior to cryopreservation in clinical conditions could increase the number of ejaculates of Purebred Spanish stallions suitable for this processing. Using colloidal centrifugation, the percentage of ejaculates available to be frozen was increased from 35% to 71%, allowing us to obtain from poor-quality fresh ejaculates thawed sperm doses with similar sperm quality to that of good-quality fresh ejaculates. These results could potentially be of great interest in the equine reproductive industry when dealing with other individuals or breeds in which, initially, low sperm quality prevents or limits their inclusion in sperm-cryopreservation programs.

**Abstract:**

The Purebred Spanish Horse, according to our clinical experience, is characterized by having a high number of stallions that do not meet the international commercial recommendations for equine-sperm cryopreservation. This means that artificial insemination with frozen semen from these stallions is less widespread than in other breeds. In this study, we investigated if the incorporation of single-layer colloidal centrifugation prior to cryopreservation in clinical conditions could increase the number of ejaculates of Purebred Spanish stallions suitable for this processing, observing the influence of centrifugation and freezing extender protocol on post-thawed sperm motility. Using colloidal centrifugation, the percentage of ejaculates available to be frozen was increased from 35% (6/17) to 71% (12/17), doubling the number of samples that could have been subjected to cryopreservation. We only found significant differences in linearity (LIN) and lateral head displacement (ALH) after 5 min of incubation at 37 °C between colloidal and simple centrifugation processing techniques. No significant differences were found between the two different colloidal protocols in any of the variables considered. Colloidal centrifugation allowed us to obtain, from worse fresh-quality ejaculates, thawed sperm doses with similar quality to that of good-quality ejaculates. BotuCrio^®^ produced, in general, higher motility parameters and its characteristics than the other extenders analyzed, with significant differences found in comparison to Inra-Freeze^®^ and Lac-Edta in both total (MOT) and progressive motility (PMOT) when using colloidal centrifugation and only in PMOT when applying simple centrifugation. Colloidal centrifugation optimized the efficiency of cryopreservation, as it allowed us to increase the number of ejaculates of Purebred Spanish Horses suitable to be frozen. Including these semen processing techniques in the freeze test could help to optimize equine-sperm cryopreservation protocols, especially when dealing with individuals or breeds for which initially low sperm quality prevents or limits their inclusion in sperm cryopreservation programs.

## 1. Introduction

Traditionally, stallions are classified as “Good/Bad Freezers” according to the capacity of their sperm to be cryopreserved [1]. Equine breeder’s selection has caused this individual variability to increase, and, nowadays, it is considered as one of the main factors that has prevented the widespread use of cryopreserved semen [2,3]. In general, 20–30% of stallions are considered to have a good frozen–thawed sperm quality, 40–60% average frozen–thawed quality and the other 20–30% are not suitable for cryopreservation [2], regardless of their fertility in natural mating [2,4,5]. This variability has also been reported between breeds [6,7]. 

In clinical practice, due to this variability, it is necessary to perform a freeze test in which one ejaculate is divided and processed with different cryopreservation protocols in order to establish which individual procedure better optimizes results [2,8]. 

There are not many papers presenting the seminal characteristics of the Purebred Spanish (P.R.E.) Horse; however, according to our clinical experience, this breed is characterized by having few stallions with more than 50% progressive-motility fresh ejaculates [9,10], which may explain why many P.R.E. stallions do not meet the international commercial recommendations for equine-sperm cryopreservation, for which percentage of progressive motility is needed to be over 50–70% in the fresh sperm before freezing [2,3,7,11] and 30–35% post-thawing [12,13]. This initial selection regarding sperm motility in raw semen is the most important variable affecting the acceptable post-thaw quality of cryopreserved ejaculates [7].

All these limitations, according to our clinical experience, determine that artificial insemination with frozen semen in P.R.E. horses is less widespread than in other breeds [14] and finding systems and procedures to increase the number of stallions and ejaculates suitable to be cooled and cryopreserved is an important goal. Among these techniques, colloidal purifying systems are proven to improve sperm quality [15,16,17,18,19,20,21] in protocols compatible with equine clinic procedures [22,23] and their use is recommended to improve fertility in sub-fertile stallions [1,24]. 

In this study, we investigated if the incorporation of two different single-layer colloidal-centrifugation protocols prior to cryopreservation in clinical conditions in the P.R.E. horse could increase the number of stallions or ejaculates suitable for this processing, observing the influence of centrifugation and freezing extender protocol on post-thawed sperm motility. 

## 2. Materials and Methods

### 2.1. Animals

A total of six P.R.E. horses, located at Centro Militar de Cría Caballar de Ávila (CCFAA) (40.6:N 4.70:W) in Spain, were used. Clinically healthy stallions, with proven fertility, ranging in age from 7 to 19 years, were kept under controlled feeding and housing conditions. Stallions were collected on a regular basis (two collections/week) after the stabilization of the extragonadal sperm reserves, which was performed at the beginning of the reproductive season in March. 

Depletion of extragonadal sperm reserves was performed by daily semen collection until the stabilization of the number of sperm in the ejaculates [25]. The number of days required differed between stallions, with a range between 4–5 consecutives days.

All stallions included in the study were subjected to an initial sperm evaluation before the beginning of the reproductive season, where morphological abnormalities could not exceed 30%.

### 2.2. Semen Collection

Semen was collected by allowing the stallions to mount a phantom and ejaculate into a Missouri-model artificial vagina (Nasco, Fort Atkinson, WL, USA), lubricated with a sterile non-spermicidal gel (IMV technologies, L´Aigle, France) and warmed to 45 to 50 °C prior to collection. A mare in estrous was used as sexual stimulation. Semen was filtered to capture gel. A total of three ejaculates per stallion were used, except from stallion D, for which only two ejaculates were processed (*n* = 17). 

### 2.3. Experimental Design

Following international commercial recommendation for equine sperm [2,3,7], those ejaculates whose progressive motility in fresh semen was higher than 60% were classified as “Suitable for Cryopreservation” (SC). These ejaculates were subjected to the standard protocol, which includes simple centrifugation. 

Ejaculates whose progressive motility in fresh semen was lower than 60% were classified as “Non-Suitable for Cryopreservation” (NSC) and were processed through colloidal centrifugation (Protocols 1 and 2).

After colloidal centrifugation, samples in which progressive motility increased ≥60% were reclassified as “Acceptable After Colloidal Centrifugation” (AACC), distinguishing between “Acceptable After Colloidal Centrifugation with Protocol 1”, (AACC1) and “Acceptable After Colloidal Centrifugation with Protocol 2” (AACC2). Semen samples whose progressive motility did not increase to 60% after colloidal centrifugation were definitely rejected for cryopreservation (non-processed samples, NP).

**Figure 1:**
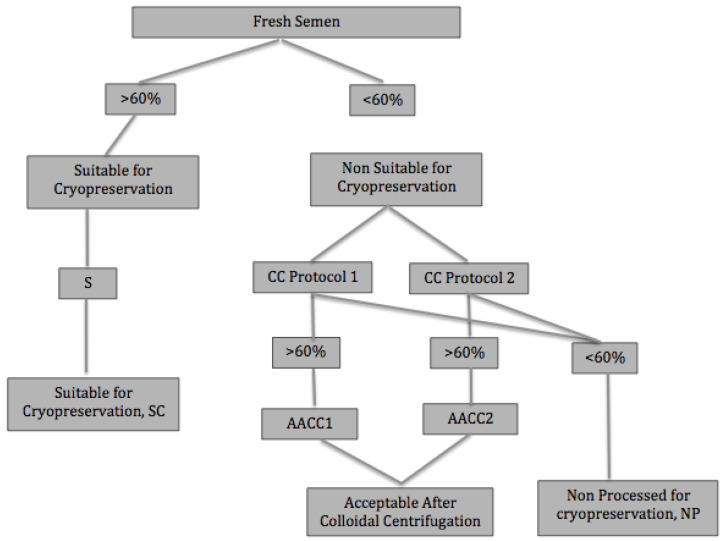
Semen centrifugation protocols. S: simple centrifugation (10 mL extended semen (1:1, *v*:*v* in Inra96^®^); 450 g for 7 min); CC: colloidal centrifugation protocol 1 (10 mL extended semen (1:1, *v*:*v* in Inra96^®^) over 10 mL of Bottom Layer^®^; 300 g for 20 min) and Protocol 2 (5 mL raw semen over 5 mL of Bottom Layer^®^; 300 g for 20 min); SC: suitable for cryopreservation; AACC: acceptable after colloidal centrifugation; AACC1: acceptable after Colloidal centrifugation with Protocol 1; AACC2: acceptable after colloidal centrifugation with Protocol 2.

Samples with ≥60% progressive motility either in fresh semen or after colloidal centrifugation (SC, AACC1 and AACC2) were included in the freeze test (Figure 1). 

### 2.4. Fresh Ejaculate Evaluation

The 17 collected, gel-free ejaculates were immediately transported to the laboratory and maintained at 37 °C for evaluation and processing. 

Spermatozoa concentration and motion characteristics were evaluated using a computer-assisted sperm-motion analyzer microscope (Sperm Class Analyzer^®^, Microptic SL, Barcelona, Spain) equipped with a heated stage and phase contrast optics (X 20 objective, Optiphot-2, Nikon, Japan). Warmed (37 °C) analysis chambers (fixed height of 20 μm) affixed to microscope slides (Leja Standard Count 2 chamber slides; Leja Products, B.V., Nienw-Vennep, The Netherlands) were loaded with 2 μL volume of extended semen (1:40, *v*:*v* in Inra96^®^). The significant settings used for CASA [22] were as follows: STR threshold for progressive motility, 60%; LIN threshold for circular spermatozoa, 50%; 32 frames per sequence; minimum of 15 frames per object; minimum area for objects 25 pix and 10 mm/s as velocity limit for immobile objects. A minimum of 500 spermatozoa were analyzed per sample. Experimental endpoints included: percentage of total motile sperm (%; MOT) and percentage of progressively motile sperm (%; PMOT). 

### 2.5. Semen Centrifugation 

Simple centrifugation: 10 mL of extended semen (1:1, *v*:*v* in Inra96^®^) in a 50 mL falcon tube and centrifuged (EBA 21 Centrifuge, Hettich^®^) at 450 g for 7 min [1].

Colloidal centrifugation: two different colloidal-centrifugation protocols were used. In 50 mL falcon tubes, 10 mL of extended semen (1:1, *v*:*v* in Inra96^®^) were pipetted and carefully layered, to avoid phase mixing, over 10 mL of Equipure Bottom Layer^®^ with a density of 80% (Nidacon, International AB, Mölndal, Sweden) (Protocol 1) or 5 mL of raw semen over 5 mL of Equipure Bottom Layer^®^ (Protocol 2). Both were equilibrated at 22 °C and centrifuged (EBA 21 Centrifuge, Hettich^®^) at 300 g for 20 min as described by Gutiérrez-Cepeda et al. [22]. 

### 2.6. After-Centrifugation Sperm Evaluation

After centrifugation, the supernatant and most of the gradient material was removed by aspiration. An aliquot of the resulting homogenized sperm pellet was added to a tube containing 1 mL of equilibrated Inra96^®^, to obtain a final concentration of 50 × 10^6^ spermatozoa/mL. 

Spermatozoa motion characteristics were evaluated using a computer-assisted sperm-motion analyzer microscope (Sperm Class Analyzer^®^, Microptic SL, Barcelona, Spain), as described in Section 2.4. Experimental endpoints included: percentage of total motile sperm (%; MOT) and percentage of progressively motile sperm (%; PMOT).

### 2.7. Freeze-Test

A total of twelve samples were, finally, processed for cryopreservation. Each sample was subjected to three different freezing protocols with three different freezing extenders commonly used in the equine reproductive industry, modified Lac-EDTA [26], Inra-Freeze^®^ (IMV technologies, L´Aigle, France) and BotuCrio^®^ (BioTech, Botucatu, Sao Paulo, Brazil). The required volume of each pellet to obtain a final concentration of 50 × 10^6^ spermatozoa/mL (25 × 10^6^ spermatozoa/per straw) was added to a fixed volume of 5mL of each extender [27]. After resuspension, semen was packed into 0.5 mL polyvinylchloride straws (IMV International, St Paul, MM, USA). Cooling rate used was that described for each extender:

- Lac-EDTA: straws were frozen directly without a cooling phase [28].
- Inra-Freeze^®^: straws were frozen after being slowly cooled to 4 °C (−0.3 °C/min) over an hour, as recommended by the manufacturer.
- BotuCrio^®^: straws were frozen after being cooled at 4 to 6 °C for 20 min, as recommended by the manufacturer.

All straws were similarly frozen horizontally in racks placed 4 cm above the surface of liquid nitrogen for 7 min, after which they were directly plunged into liquid nitrogen [29].

### 2.8. Post-Thaw Sperm Evaluation

After 4 weeks of storage, straws were thawed by immersion in a 37 °C water bath for 1 min. Spermatozoa motion characteristics were evaluated using a computer-assisted sperm-motion analyzer microscope (Sperm Class Analyzer^®^, Microptic SL, Barcelona, Spain), as described in Section 2.4. Experimental endpoints included: percentage of total motile sperm (%; MOT); percentage of progressively motile sperm (%; PMOT); curvilinear velocity (μm/s; VCL); straight-line velocity (μm/s; VSL); average path velocity (μm/s; VAP); linearity (%; LIN); straightness (%; STR); amplitude of lateral displacement (μm; ALH) and beat cross frequency (Hz; BCF).

A minimum of 500 spermatozoa per sample were counted. The sperm was kept at 37 °C before analysis performed both 5 and 30 min after thawing.

### 2.9. Statistical Analysis

The effect of centrifugation and freezing extender treatment on the different motility variables were determined by ANOVA and Duncan Tests, both in general and within each group (SC and AACC), independently, for the centrifugation and the freezing extender treatments. The effect of the stallion variability was considered in the analysis. Significance was set at *p* < 0.05. Data were processed using the SPSS-19 statistical package. 

## 3. Results

### 3.1. Prior Cryopreservation Evaluation

Progressive motility values for fresh semen and after centrifugation protocols are shown in Table 1.

From the total of seventeen ejaculates, six presented PMOT > 60% in fresh sperm and were classified as SC. The other eleven ejaculates presented fresh PMOT < 60% and were included as NSC and subjected to colloidal centrifugation (Protocol 1 and 2). From these eleven NSC ejaculates, six obtained enough progressive motility (PMOT > 60%) to be included in the freeze test and were reclassified as AACC. 

Five ejaculates did not obtain enough progressive motility (PMOT < 60%) to be frozen and were rejected for cryopreservation, NP.

Therefore, a total of fourteen samples from twelve ejaculates were processed for cryopreservation, three samples (1SC, 2SC, 3SC) from three ejaculates (1, 2 and 3) of stallion A, one sample (1SC) from one ejaculate (1) of stallion B, three samples (1SC, 3AACC1, 3AACC2) from two ejaculates (1 and 3) of stallion C, two samples (1AACC1, 2AACC1) from two ejaculate (1 and 2) of stallion D, one sample (1AACC2) from one ejaculate (1) of stallion and four samples (1SC, 2AACC1, 3AACC1 y 3AACC2) from three ejaculates (1, 2 and 3) of stallion F.

Among the six stallions included in the study, only stallion A presented enough fresh quality to process his three ejaculates. For 64.7% of the ejaculates, single-layer colloidal centrifugation with Equipure^®^ was necessary to try to improve semen quality. Three ejaculates from stallion A and one from stallions B, C and F were cryopreserved following simple centrifugation, while colloidal centrifugation was required to freeze two ejaculates from stallion E, two ejaculates from F and one from stallions C and D.

### 3.2. Post-Thaw Evaluation

When studying freezing extenders and centrifugation protocols, we observed significant differences between horses (*p* < 0.05) in all variables considered. The effect of stallion was then considered when analyzing statistically the effect of the different treatments of the study. However, these differences between stallions were reduced within the SC and AACC groups.

Differences in post-thawing values after both 5 and 30 min of incubation between groups are shown in Table 2. There were only significant differences in variables LIN5, where AACC obtained higher values, and in ALH5, where SC presented a significant increased. 

Subsequently, differences between freezing extenders were observed in post-thawing motility parameters within the SC group (Table 3). 

In the SC group, the BotuCrio^®^ freezing extender produced significantly higher values than the others in PMOT and BCF both 5 and 30 min and in VSL 5 min after thawing. When considering velocity parameters, both BotuCrio^®^ and Lac-EDTA were higher than Inra-Freeze^®^, with significant differences found 5 min after thawing in VCL and 30 min after thawing in VCL, VSL and VAP. 

Table 4 represents the differences between freeze-test extenders in post-thawing motility parameters within AACC groups. 

Regarding the AACC group, MOT and PMOT, both 5- and 30-minute post-thawing were significantly higher (*p* < 0.05) with BotuCrio^®^ than with the other extenders considered. STR and LIN 5 min after thawing were significantly lower with Lac-EDTA than with the other extenders. 

Mean post-thawed progressive motility values (%) for each stallion and freezing extender are represented in Table 5.

## 4. Discussion

International commercial recommendations for equine-sperm cryopreservation stipulates that the percentage of progressive motility must be over 50–70% in the fresh sperm before freezing [2,3,7,11] and 30–35% post-thawing [2,12,13]. These minimal requirements for the raw-semen quality of conventional equine-semen freezing programs are needed to obtain a good post-thawing sperm quality, and, among them, initial sperm motility is proven to be the most important affecting variable [7]. In our study, when seminal characteristics were analyzed in the seventeen fresh ejaculates (Table 1), we observed that only nine of the seventeen ejaculates (52.94%) presented a progressive motility greater than 50% and only six over 60% (35%). Although Miró and Papas found no differences in some sperm-quality parameters of both fresh and frozen/thawed samples between P.R.E. and non-P.R.E. stallions [30], our study showed, as also previously described [9,10], that not many ejaculates from P.R.E. stallions meet these international commercial standards. 

Between the eleven ejaculates labelled “NSC” (PMOT<60%), when subjected to colloidal-centrifugation protocols prior to cryopreservation, six obtained sufficient motility to be included in the freeze test (AACC), increasing from 35% (6/17) to 71% (12/17) the percentage of ejaculates available to be frozen. Protocol 1 was more effective clinically, as it improved five out of six of the “NSC”ejaculates, whereas Protocol 2 only optimized three. 

Differences between ejaculates of the same stallion were observed. This variability in the sperm motility observed between the ejaculates of the same stallion could be explained by the influence of factors such as seminal plasma composition and volume, which is also affected by sexual stimulation and sperm volume [31,32].

The importance of stallion selection prior to colloidal-centrifugation cryopreservation protocols was described by Mancill et al. [17] and Hoogewijs et al. [33]. In concordance with them, we did not process good-quality fresh ejaculates through colloidal centrifugation. Our study demonstrated that the clinical incorporation of colloidal centrifugation in low-quality ejaculates in cryopreservation protocols is of great interest, as it increases the efficiency of the freezing technique, as the number of ejaculates suitable for cryopreservation in each stallion is increased and it allows obtaining thawed sperm doses from stallions that would otherwise be rejected for cryopreservation according to its fresh sperm quality.

No significant differences were found in post-centrifugation sperm quality between the two different colloidal protocols used. When comparing post-thawed sperm quality between centrifugation protocols, we only found significant differences in LIN and ALH after 5 min of incubation.

What is more, colloidal centrifugation has proved to increase equine [15,16,17,20,34], dog [35,36,37] and buck [38] sperm quality when applied to thawed samples. Various authors [16,26,38,39,40,41,42,43] state that colloidal centrifugation prior to freezing also increases the cryosurvival of spermatozoa and/or post-thawed sperm quality. Macías García et al. [44] stated that colloidal centrifugation selects a sperm subpopulation that responds differently to osmotic shock, helping to withstand the cryopreservation process [43]. This fact could be related to lower reactive oxygen species’ (ROS) production and contamination which, in other studies, have been proven to be achieved after colloidal centrifugation [30,43].

Hoogewijs et al. [33] found an improvement in quality both before and after cryopreservation compared to cushion techniques when applying colloidal centrifugation prior to cryopreservation. This effect was higher in stallions classified as “non-suitable for cryopreservation” (stallions that produced post-thawing progressive motility <30%) [40]. In another study, Hoogewijs et al. [40] also found that when comparing these two selecting techniques prior to cryopreservation, colloidal centrifugation allowed the maintenance of the improvement after 120 min of incubation at 37 °C. Hidalgo et al. [42] also showed that single-layer centrifugation prior to cryopreservation allowed obtaining higher sperm motility and lower DNA fragmentation after thawing than when applying a modified colloid swim up and/or a sperm washing procedure. In addition, El-Essawe et al. [43] described beneficial effects on sperm chromatin integrity, high mitochondrial membrane potential and a reduction in hydrogen-peroxide production after thawing.

Similarly, Mancill et al. [17] found a higher thawing sperm quality in “subfertile stallions” when colloidal centrifugation was performed prior to cryopreservation compared to simple centrifugation, although no differences were found between centrifugation techniques within the fertile group. Mancill et al. [17] determined that the incorporation of colloidal centrifugation prior to cryopreservation in fertile stallions is not effective, as it includes an additional processing step, which causes a significant loss of presumably normal sperm and unnecessarily increases economic and technical costs.

The present colloidal-centrifugation protocols have already been used in previous works without finding a negative impact on outcomes [22,27]. However, as mentioned before, in the present work, Protocol 1 was more effective at improving the quality of NSC ejaculates. This could be related to the higher sperm concentration applied over the colloid in Protocol 2 (raw sperm), as has been previously described [45].

When studying freezing extenders and centrifugation protocols, there were significant differences between horses (*p* < 0.05) in all variables considered, which reflects the characteristic individual sperm-response variability among the horse population [2].

Regarding our results for freezing extenders, we can determine that BotuCrio^®^ produced, in general, higher values in motility parameters and their characteristics compared to the others both in the SC and AACC groups. It significantly improved progressive motility both immediately and after 30 min of incubation. Lac-EDTA showed higher results for velocity parameters (VCL, VSL and VAP) than Inra-Freeze^®^ but only within the SC group. Studies in dogs [46] and boars [47] have revealed that high sperm velocities are landmarks of fertility both in vivo and in vitro. It has also been reported previously that VCL is of key importance for the formation of the sperm reservoir at the utero-tubal junction in mice [48], that VCL and VAP are linked to the ability of ram spermatozoa to penetrate cervical mucus [49], and that post-thaw VSL is related to the fertility of bull [50] and human [51] spermatozoa. Likewise, some authors [46,52] described a reduction in the sperm’s ability to travel along the uterus and arrive at the fertilization site when velocity variables decreased.

These differences could be related with the cryoprotectants combination presented in the extender. Both Inra-Freeze^®^ and Lac-EDTA extenders include Glycerol as the only cryoprotectant agent, while BotuCrio^®^ includes a lower Glycerol concentration and Metilformamine. It has been published that sperm cryopreservation with amines as cryoprotectants are effective at improving motility, viability in the female genital tract and fertility of frozen–thawed sperm, as well as at reducing individual and breed variability, mainly in horses classified as “bad freezers” [6,53,54,55]. Similarly, lower cryoprotectant concentrations benefits thawed-sperm viability, especially in “bad freezers” stallions [56].

The development of new freezing extenders, as seemed to happen with amides, in combination with sperm selection techniques can be used in equine reproduction clinics to optimize sperm cryopreservation, especially when dealing with low fresh-sperm quality breeds, which limits their inclusion in freezing commercial protocols.

## 5. Conclusions

The clinical use of colloidal centrifugation on equine sperm freeze test in P.R.E. stallions optimized the efficiency of the cryopreservation technique, as low-quality ejaculates were incorporated into this procedure. Colloidal centrifugation allowed us to obtain from poor-quality fresh ejaculates thawed-sperm doses with similar sperm quality to that of good-quality fresh ejaculates. These results could potentially be of great interest in the equine reproductive industry when dealing with other individuals or breeds in which initially low sperm quality prevents or limits their inclusion in sperm cryopreservation programs. Further studies with a larger sample size are needed to confirm these results.

## Figures and Tables

**Table 1:** Progressive motility values for fresh semen and after centrifugation protocols.

Stallion	Age (Years)	Ejaculate	Group	Fresh	After Centrifugation Protocols		
					S	Protocol 1 CC	Protocol 2 CC
		1	SC	**76.9**	67.0	-	-
A	9	2	SC	**70.8**	59.2	-	-
		3	SC	**68.4**	67.9	-	-
		1	SC	**69**	69.4	-	-
B	17	2	NP	44.6	-	33.1	42.6
		3	NP	42.1	-	49.2	50.7
		1	SC	**67.2**	59.3	-	-
C	15	2	NP	38.1	-	12.4	24.7
		3	AACC1-2	56.4	-	75.7	58.4
D	9	1	AACC1	41.9	-	65.4	53.6
		2	AACC1	41.9	-	65.4	53.6
		1	AACC2	56.5	-	54.3	66.8
E	19	2	NP	49.6	-	49.0	48.5
		3	NP	49.7	-	41.8	32.0
		1	SC	**71.2**	52.3	-	-
F	7	2	AACC1	46.3	-	61.8	49.8
		3	AACC1-2	52.1	-	60.8	64.3

Progressive motility (%) for fresh semen (Fresh), simple (S (10 mL extended semen), 1:1, *v*:*v* with Inra96^®^) or colloidal centrifugation (Protocol 1 CC (10 mL extended semen, 1:1, *v*:*v* with Inra96^®^, over 10 mL bottom layer^®^) and Protocol 2 CC (5 mL raw semen over 5 mL bottom layer^®^)). Group: suitable for cryopreservation (SC, bold numbers), acceptable after colloidal centrifugation (underlaying numbers) with Protocol 1 (AACC1) or Protocol 2 (AACC2) or both (AACC1-2) and non-processed (NP).

**Table 2:** Motility parameters values 5 and 30 min after thawing.

GROUP	SC	AACC1	AACC2
MOT5	59.10 ^a^ ± 20.88	51.55 ^a^ ± 14.88	44.22 ^a^ ± 15.11
PMOT5	45.59 ^a^ ± 17.31	40.29 ^a^ ± 17.09	38.51 ^a^ ± 12.82
VCL5	84.08 ^a^ ± 14.61	76.54 ^a^ ± 12.94	81.01 ^a^ ± 11.69
VSL5	59.71 ^a^ ± 13.67	62.43 ^a^ ± 10.54	68.88 ^a^ ± 7.45
VAP5	69.02 ^a^ ± 13.39	68.93 ^a^ ± 11.45	73.39 ^a^ ± 8.50
LIN5	71.99 ^b^ ± 8.68	81.87 ^a^ ± 6.55	84.54 ^a^ ± 6.82
STR5	88.89 ^a^ ± 5.54	90.75 ^a^ ± 5.56	93.66 ^a^ ± 3.10
ALH5	2.58 ^a^ ± 0.54	1.84 ^b^ ± 0.28	2.07 ^b^ ± 0.30
BCF5	10.53 ^a^ ± 1.69	8.51 ^a^ ± 1.29	9.21 ^a^ ± 0.96
MOT30	50.28 ^a^ ± 15.09	41.57 ^a^ ± 16.01	37.80 ^a^ ± 14.87
PMOT30	36.91 ^a^ ± 11.90	31.01 ^a^ ± 16.46	27.42 ^a^ ± 14.28
VCL30	78.24 ^a^ ± 12.57	74.79 ^a^ ± 14.30	69.44 ^a^ ± 14.42
VSL30	59.79 ^a^ ± 9.19	60.16 ^a^ ± 11.18	58.00 ^a^ ± 13.43
VAP30	66.33 ^a^ ± 10.66	65.76 ^a^ ± 13.09	62.23 ^a^ ± 14.10
LIN30	76.82 ^a^ ± 7.29	80.75 ^a^ ± 7.38	83.54 ^a^ ± 7.71
STR30	90.37 ^a^ ± 4.59	91.67 ^a^ ± 3.90	93.21 ^a^ ± 4.16
ALH30	2.33 ^a^ ± 0.59	1.95 ^a^ ± 0.60	1.83 ^a^ ± 0.41
BCF30	9.76 ^a^ ± 1.45	8.71 ^a^ ± 1.34	8.48 ^a^ ± 1.67

Means values (mean ± SD) of percentage of total motile sperm (%; MOT); percentage of progressively motile sperm (%; PMOT); curvilinear velocity (μm/s; VCL); straight-line velocity (μm/s; VSL); average path velocity (μm/s; VAP); linearity (%; LIN); straightness (%; STR); amplitude of lateral displacement (μm; ALH) and beat cross frequency (Hz; BCF), after 5 (5) and 30 (30) minutes of incubation. Centrifugation protocol: SC (simple centrifugation; 10 mL extended semen), AACC1 (Protocol 1: 10 mL extended semen over 10 mL bottom layer^®^) and AACC2 (Protocol 2: 5 mL raw semen over 5 mL bottom layer^®^). Superscript letters represent significant differences between treatments (*p* < 0.05) in each variable.

**Table 3:** Motility parameters 5 and 30 min after thawing for freezing extenders in SC group.

EXTENDER	Botucrio^®^	Inra-Freeze^®^	Lac-Edta
MOT5	73.48 ^a^ ± 5.71	57.33 ^a^ ± 18.71	46.45 ^a^ ± 25.63
PMOT5	61.70 ^a^ ± 6.49	42.17 ^b^ ± 11.01	32.90 ^b^ ± 17.72
VCL5	93.24 ^a^ ± 13.10	71.38 ^b^ ± 7.07	87.61 ^a^ ± 13.99
VSL5	66.40 ^a^ ± 6.86	53.46 ^b^ ± 4.30	59.26 ^a,b^ ± 4.75
VAP5	72.49 ^a^ ± 6.05	63.88 ^a^ ± 10.02	70.70 ^a^ ± 7.70
LIN5	72.45 ^a^ ± 13.12	75.07 ^a^ ± 3.71	68.47 ^a^ ± 6.61
STR5	91.60 ^a^ ± 4.01	90.89 ^a^ ± 3.73	84.20 ^b^± 5.86
ALH5	2.76 ^a^ ± 0.81	2.41 ^a^ ± 0.32	2.55 ^a^ ± 0.38
BCF5	11.72 ^a^ ± 2.26	9.99 ^b^ ± 0.95	9.85 ^b^ ± 1.05
MOT30	59.27 ^a^ ± 12.90	50.66 ^a^ ± 11.84	40.92 ^a^ ± 15.36
PMOT30	46.48 ^a^ ± 12.29	33.65 ^b^ ± 6.61	30.60 ^b^ ± 10.78
VCL30	85.67 ^a^ ± 9.34	65.51 ^b^ ± 14.02	80.55 ^a^ ± 8.20
VSL30	64.54 ^a^ ± 6.16	51.61 ^b^ ± 9.31	63.23 ^a^ ± 6.37
VAP30	69.88 ^a^ ± 5.78	57.73 ^b^ ± 12.38	71.38 ^a^ ± 8.07
LIN30	75.92 ^a^ ± 9.71	75.92 ^a^ ± 7.03	78.63 ^a^ ± 5.56
STR30	92.33 ^a^ ± 3.15	90.02 ^a^ ± 6.04	88.75 ^a^ ± 4.15
ALH30	2.59 ^a^ ± 0.67	2.16 ^a^± 0.59	2.23 ^a^ ± 0.49
BCF30	10.96 ^a^ ± 1.16	9.20 ^b^ ± 1.57	9.13 ^b^ ± 0.86

Means values (mean ± SD) of percentage of total motile sperm (%; MOT); percentage of progressively motile sperm (%; PMOT); curvilinear velocity (μm/s; VCL); straight-line velocity (μm/s; VSL); average path velocity (μm/s; VAP); linearity (%; LIN); straightness (%; STR); amplitude of lateral displacement (μm; ALH) and beat cross frequency (Hz; BCF), after 5 (5) and 30 (30) minutes of incubation. Superscript letters represent significant differences between treatments (*p* < 0.05) in each variable.

**Table 4:** Motility parameters 5 and 30 min after thawing for freezing extenders in AACC group.

EXTENDER	Botucrio^®^	Inra-Freeze^®^	Lac-Edta
MOT5	64.14 ^a^ ± 6.79	43.59 ^b^ ± 11.30	38.30 ^b^ ± 13.46
PMOT5	55.69 ^a^ ± 11.76	34.43 ^b^ ± 10.04	27.11 ^b^ ± 8.16
VCL5	79.62 ^a^ ± 8.86	71.90 ^a^ ± 16.64	82.54 ^a^ ± 9.29
VSL5	66.31 ^a^ ± 11.29	62.39 ^a^ ± 15.22	64.27 ^a^ ± 9.67
VAP5	70.94 ^a^ ± 10.90	66.11 ^a^ ± 15.30	73.53 ^a^ ± 9.39
LIN5	83.00 ^a^ ± 7.83	86.61 ^a^ ± 2.36	77.56 ^b^ ± 6.13
STR5	93.34 ^a^ ± 3.90	94.16 ^a^ ± 2.01	87.43 ^b^ ± 6.12
ALH5	1.96 ^a^ ± 0.27	1.90 ^a^ ± 0.39	1.94 ^a^ ± 0.28
BCF5	9.26 ^a^ ± 1.08	8.54 ^a^ ± 1.58	8.63 ^a^ ± 0.83
MOT30	54.03 ^a^ ± 5.96	35.86 ^b^ ± 17.09	29.80 ^b^ ± 10.85
PMOT30	42.03 ^a^ ± 11.30	23.49 ^b^ ± 17.22	22.19 ^b^ ± 10.01
VCL30	72.93 ^a^ ± 10.70	65.76 ^a^ ± 10.49	79.95 ^a^ ± 19.75
VSL30	61.63 ^a^ ± 11.59	54.01 ^a^ ± 10.44	61.79 ^a^ ± 13.85
VAP30	65.95 ^a^ ± 11.76	57.18 ± 10.09	69.81 ^a^ ± 16.67
LIN30	84.35 ^a^ ± 8.82	82.03 ^a^ ± 6.57	78.01 ^a^ ± 6.63
STR30	93.43 ^a^ ± 4.09	94.23 ^a^ ± 2.42	88.70 ^a^ ± 3.55
ALH30	1.80 ^a^ ± 0.40	1.85 ^a^ ± 0.60	2.11 ^a^ ± 0.64
BCF30	8.46 ^a^ ± 1.03	9.00 ^a^ ± 0.79	8.44 ^a^ ± 2.12

Means values (mean ± SD) of percentage of total motile sperm (%; MOT); percentage of progressively motile sperm (%; PMOT); curvilinear velocity (μm/s; VCL); straight-line velocity (μm/s; VSL); average path velocity (μm/s; VAP); linearity (%; LIN); straightness (%; STR); amplitude of lateral displacement (μm; ALH) and beat cross frequency (Hz; BCF), after 5 (5) and 30 (30) minutes of incubation. Superscript letters represent significant differences between treatments (*p* < 0.05) in each variable.

**Table 5:** Mean progressive motility values (%) 5 min after thawing for freezing extenders and stallion.

Stallion	Number of Frozen Ejaculates	Botucrio^®^	Inra-Freeze^®^	Lac EDTA
Stallion A	3	62.67	42.83	17.77
Stallion B	1	65.8	46.4	39.7
Stallion C	2	64.77	32.97	34.17
Stallion D	2	55.6	33.3	25.6
Stallion E	1	39.6	36.2	30.3
Stallion F	3	54.2	38.75	36.8

## Data Availability

Not applicable.
